# Multiple Phenotypes Resulting from a Mutagenesis Screen for Pharynx Muscle Mutations in *Caenorhabditis elegans*

**DOI:** 10.1371/journal.pone.0026594

**Published:** 2011-11-02

**Authors:** Andrew Ferrier, Alexandra Charron, Yama Sadozai, Lynn Switaj, Anneliese Szutenbach, Pliny A. Smith

**Affiliations:** Biology Department, Lake Forest College, Lake Forest, Illinois, United States of America,; Centre for Genomic Regulation, Spain

## Abstract

We describe a novel screen to isolate pharyngeal cell morphology mutants in *Caenorhabditis elegans* using *myo-2::GFP* to rapidly identify abnormally shaped pharynxes in EMS (Ethyl Methanesulfonate) mutagenized worms. We observed over 83 *C. elegans* lines with distinctive pharyngeal phenotypes in worms surviving to the L1 larval stage, with phenotypes ranging from short pharynx, unattached pharynx, missing cells, asymmetric morphology, and non-adherent pharynx cells. Thirteen of these mutations have been chromosomally mapped using Single Nucleotide Polymorphisms (SNPs) and deficiency strain complementation. Our studies have focused on genetically mapping and functionally testing two phenotypes, the short pharynx and the loss of muscle cohesion phenotypes. We have also identified new alleles of *sma-1*, and our screen suggests many genes directing pharynx assembly and structure may be either pharynx specific or less critical in other tissues.

## Introduction

The *C. elegans* pharynx exhibits progressive restriction of cell fate during development, ultimately resulting in the expression of differentiation factors and structural proteins essential to its function as a neuromuscular pump [1], [2], [3]. Seven different cell types are specified during pharynx organogenesis; and within these cell types, sub-specialization occurs producing distinct anterior to posterior characteristics [4]. For example, eight different classes of pharynx muscle differ in morphology, producing the distinct bi-lobed pharynx that enables the worm to pump bacteria from the environment and pulverize this food before it passes into the intestine.

In *C. elegans*, *pha-4* is an organ identity gene involved in the specification and differentiation of all cells destined to become the pharynx [5], [6], [7]. If *pha-4* expression is eliminated through mutation or RNA interference, the entire pharynx fails to develop; ectopic expression of *pha-4* in early embryos converts additional cells to become pharynx cells [5], [8]. The *pha-1* gene allows for initial development of pharyngeal precursor cells, but then affects differentiation of all pharynx cells types after the 1½-fold stage of embryogenesis when differentiation markers such as pharyngeal myosin and intermediate filaments are normally activated [9]. While less dramatic, mutations in *glp-1*, *tbx-35*, or *lag-1* result in a loss of all pharynx cells derived from ABa or MS lineage, resulting in formation of a half pharynx. In the cases of *pha-4*, *glp-1*, *tbx-35*, and *lag-1*, the loss of pharynx cells is not cell-type specific, rather entire regions of the pharynx are deleted such as the anterior ABa derived-pharynx in *glp-1* mutants [5], [8], [10], [11], [12], [13].

Multiple genes have been identified that are expressed in distinct pharyngeal cell types, such as *myo-2* and *ceh-22* in pharynx muscle and intermediate filaments in marginal cells; however only *tbx-2* is essential to specify a particular cell fate, in this case, anterior ABa derived pharynx muscle cells [6], [14], [15], [16], [17], [18]. Interestingly, the posterior sets of pharynx muscle cells derived from the MS blastomere form normally in the absence of TBX-2 and non-muscle ABa derived pharynx does not appear to require TBX-2 function [14]. No gene has been found that is specifically required for posterior pharynx muscle specification. Many previously described pharynx genes have been found using genetic screens, including alleles of genes *pha-1*, *pha-2*, *pha-3*, and *pha-4*; however, these screens were not optimized for screening of morphological changes in pharynx muscle [5], [9], [19]. The ability to use Green Fluorescent Protein (GFP) expressed specifically in pharynx muscle of larva has simplified the screening for mutations that affect pharynx structure in live worms. Thus, the initial goal of our mutagenesis screen was to isolate a gene necessary for either all pharynx muscle, or posterior pharynx muscle specification; however, we instead found phenotypes that ranged from a short pharynx to pharynxes that are barely distinguishable.

This report describes multiple classes of mutant pharynx phenotypes isolated from an Ethyl Methanesulfonate (EMS) mutagenesis screen for larvae with phenotypes such as short pharynx, thin-cylindrical pharynx, non-adherent cells, anterior pharynx absent, pharynx cells outside the basement membrane, and pharynx unattached. This report provides an overview of the results of the screen, and focuses on the mapping and characterization of short pharynx mutants and non-adherent muscle cell pharynx mutants.

## Results

**Figure 1:**
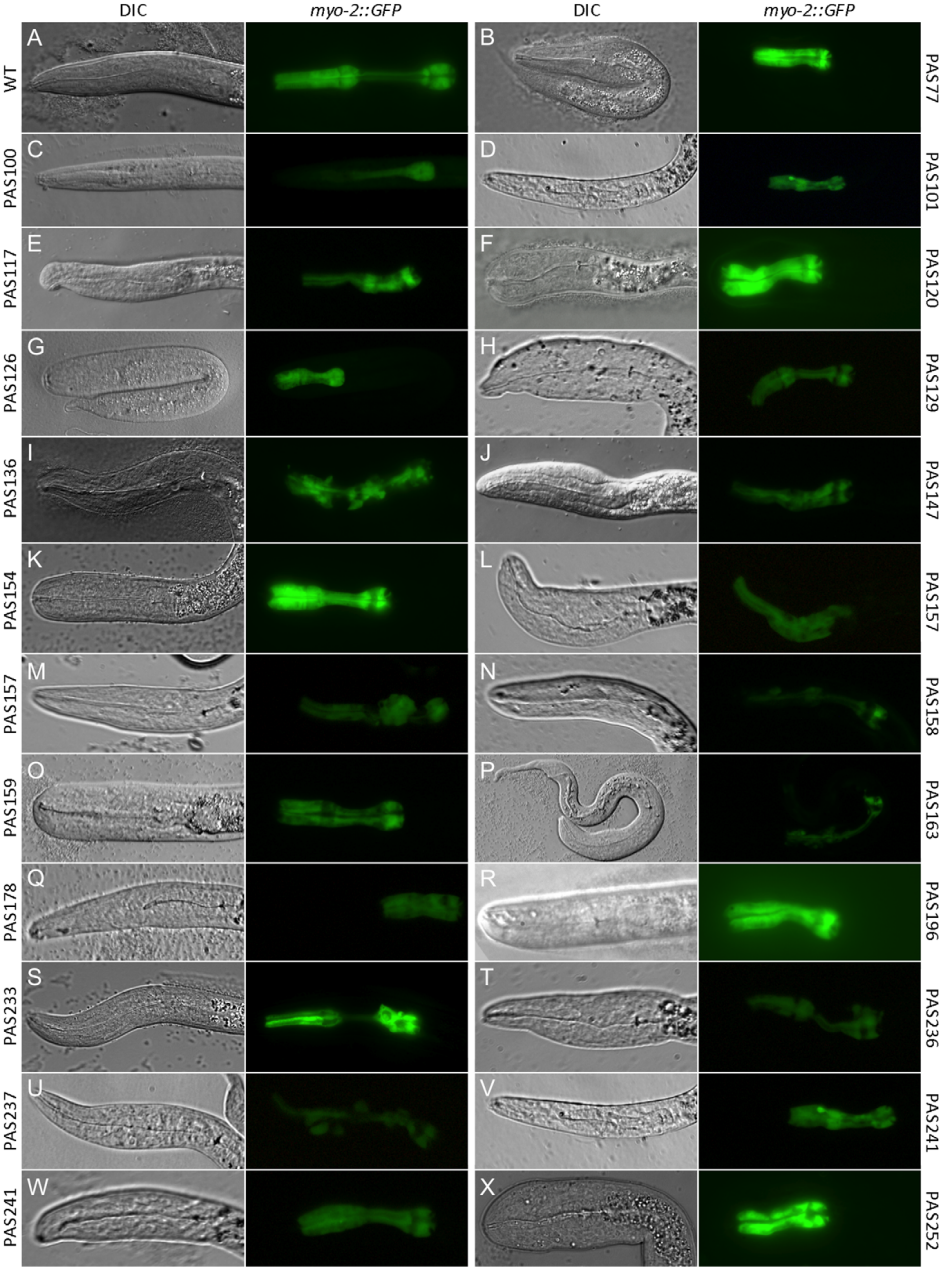
Variety of phenotypes observed from EMS mutagenesis screen. Brightfield/DIC columns 1 and 3; GFP columns 2 and 4. (A) PD4792 wild-type phenotype with distinct procorpus, anterior bulb, isthmus, and posterior bulb. (B) PAS77 short pharynx phenotype. (C) PAS100 thin pharynx with less anterior GFP expression than wild type. (D) PAS101 pharynx unattached. (E) PAS117 anterior bulb diminished in size. (F) PAS120 short pharynx and bulbous head. (G) PAS126 short pharynx. (H) PAS129 short pharynx with head defects. (I) PAS136 pharynx muscle cells do not adhere to each other. (J) PAS147 cylindrical pharynx with diminished anterior bulb. (K) PAS154 short pharynx phenotype. (L and M) PAS157 pharynx asymmetry with indistinct isthmus and anterior bulb. (N) PAS158 Diminished GFP expression and asymmetric anterior pharynx. (O) PAS159 short pharynx. (P) PAS163 disorganized pharynx with unattached GFP-expressing cells. (Q) PAS178 Pun or Aph phenotype. (R) PAS196 short pharynx. (S) PAS233 larva with posterior cell displacement. (T) PAS236 short isthmus and posterior defects. (U) PAS237 pharynx muscle cells unattached. (V) PAS241 Pun phenotype. (W) PAS241 short pharynx. (X) PAS252 short pharynx.

We performed an EMS mutagenesis screen of ∼10,000 haploid genomes to identify the genes affecting posterior muscle fate in *C. elegans*, using a *myo-2::GFP* reporter to visualize pharynx morphology in L1s. Initially, the low-copy number *myo-2::GFP* (AZ217) integrated reporter strain was used in mutagenesis; however, the strain's weak fluorescence made rapid identification of pharynx abnormalities difficult under an epifluorescent stereomicroscope. Substitution of AZ217 with the more robust *myo-2::GFP* fluorescence of PD4792 made identification of mutant phenotypes more reliable; the *pes-10::GFP* expression was only seen in early embryos and we did not observe the gut-specific enhancer GFP in larvae or adults (Figure 1A, B). In total, we identified 83 possible pharynx defective strains suggestive of abnormalities in cell adhesion, cell fate, cell morphology, and migration in both anterior and posterior pharynx regions (Table 1). SNP mapping of thirteen different lines shows phenotypic alleles are present throughout the genome (Table 2). All mutant lines isolated demonstrated recessive phenotypes and behaved as single alleles. Interestingly, we did not discover any obvious posterior pharyngeal phenotypes in which MS-derived muscle was missing; however, many of the observed phenotypes appear to be unreported.

**Table 1:** Summation of pharynx phenotypes.

Phenotype*	Number
Anterior bulb morphology	18
APH (anterior pharynx absent)	5
Pharynx basement membrane defect	11
Short pharynx	20
GFP-expressing cells outside of pharynx	14
Isthmus defect	11
Pha (pharynx absent)	1
Pharynx asymmetric	21
Pharynx GFP weak/nearly absent	6
Posterior bulb defect	9
Procorpus defect	29
Pun (Pharynx unattached)	7
Thin, cylindrical pharynx	25

* Worm strains may have overlapping phenotypes.

**Table 2:** Chromosomal linkage of 13 isolated mutant strains.

Mutant Strain	Linkage	Phenotype
PAS77	LG. III	Short pharynx
PAS100	LG. X	Anterior bulb morphology
PAS101	LG. III	Pun
PAS120	LG. IV	Short pharynx
PAS132	LG. V	Thin, cylindrical pharynx
PAS136	LG. I	Amorphous pharynx
PAS138	LG. I	Amorphous pharynx
PAS154	LG. IV	Short pharynx
PAS170	LG. III	Posterior bulb defects
PAS192	LG. IV	Anterior pharynx defects
PAS202	LG. III	Short pharynx/thin pharynx
PAS252	LG. X	Short pharynx
PAS262	LG. X	Short pharynx

### The short-pharynx phenotypes

We identified 20 mutant lines that shared a similar phenotypic trait, a short pharynx and rounded mouth similar to mutant strain PAS77; nine are shown in Figure 1 (Figure 1B, F, G, H, K, O, R, W, X). Some of these short pharynx mutants were viable to the adult stage, while others died during larval development; four of the short pharynx stains have been chromosomally mapped (Table 2). Most had additional body defects, including a fat, round head.

We have attempted characterization of the stain PAS77, which has a rounded mouth and short, dumpy phenotype; the larva often arresting during the L1 stage of development. Unlike wild-type worm pharynxes which are exhibit clear distinction between the procorpus, anterior bulb, metacarpus, and terminal bulb, PAS77 has indistinguishable anatomical regions, where the terminal bulb is present but lacks the distinguishable metacarpus and procorpus (Figure 1B). In addition, the isthmus in PAS77 is much shorter compared to wild type. PAS77 homozygous worms are sterile, often arresting at the L1 stage; worms that escape arrest have a Dpy phenotype. Raising the worms on plates supplemented with 150 mM ethanol results in a slightly elevated percentage of worms escaping L1 arrest, but not significantly (12% of control worms escape, n = 250; 15% of ethanol treated).

**Figure 2:**
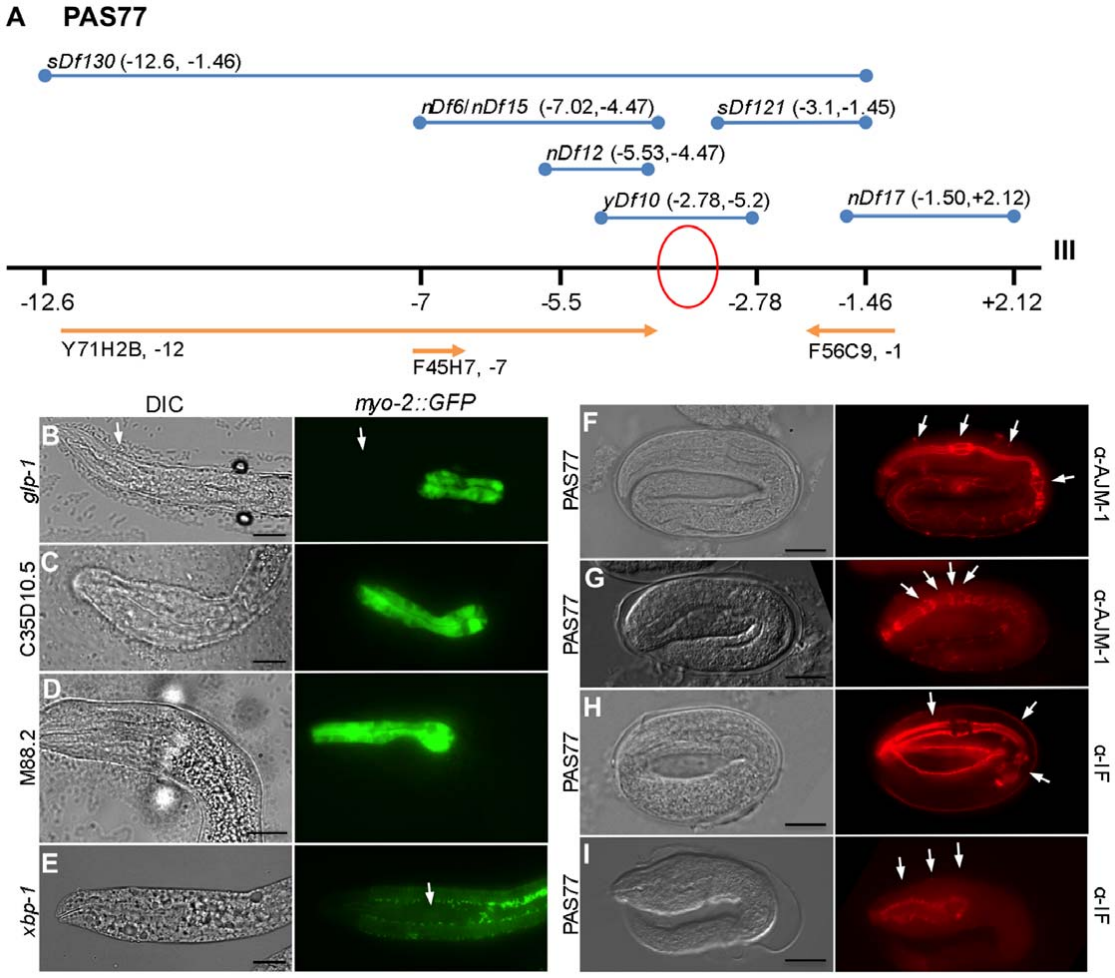
PAS77 mapping and pharynx markers. (A) Probable location of the PAS77 pharynx phenotype allele is between −4.47 cM and −3.1 cM relative to the genetic center of LG.III (red circle) derived by mapping with *DraI* specific SNPs corresponding to DNA clones Y71H2B, F45H7, and F56C9 (orange lines) and complementation with deficiency strains with overlapping chromosomal deletions (blue lines). (B) *glp-1* RNAi resulted in an Aph phenotype in >50% of larvae; serving as a control for RNAi effectiveness. Arrow shows region missing anterior pharynx cells. (C and D) C35D10.5 RNAi or M88.2 RNAi results in a short pharynx phenotype. (E) *xbp-1* (R74.3) RNAi eliminates GFP expression in pharynx. (F) wild-type MH27 AJM-1 adherens junction antibody staining shows four distinct regions in the pharynx (arrows). (G) PAS77 MH27 antibody staining shows four compressed pharynx regions (arrows). (H) Wild-type MH4 Intermediate Filament antibody staining showing three sets of marginal cells (arrows). (I) PAS77 MH4 antibody staining showing less distinct marginal cell boundaries (arrows). Bar is ∼10 µM.

Chromosomal and Interval SNP mapping with polymorphisms on Chromosome III placed the PAS77 mutant allele between genetic regions −5 and −3 m.u. relative to the center of the Linkage Group III (LG.III) (Figure 2A, orange lines). To further narrow the range of possible loci for PAS77, we completed genetic complementation with the following strains having chromosomal deletions between the genetic regions of −12.6 cM and −1.46 cM from the center of LG.III: BC4637, BC4697, CB4681, MT690, MT696, MT699, NG2618, and TY1353 (Figure 2A, blue lines). All genetic crosses produced males in the F1 generation and mutant phenotypes in the F2 generation; however, only the NG2618 cross-produced the PAS77 phenotype in the F1 generation. The non-complementation of NG2618, along with the complementation of the adjacent deficiency strains MT696 and MT696 refines the genetic mapping on the near left arm of Chromosome III, −4.47 cM and −2.78 cM from the center of LG.III.

Interestingly, the PAS77 mutant allele mapped nearby a gene named *mor-1*, mapping at III: 1.64+/−1.55 cM (WormBase release 226), although two previous releases of WormBase have shown *mor-1* mapping ∼5 cM and ∼2 cM to the left of its current predicted genetic position (WormBase Release WS180: III:−6.45+/−5.225 cM; WS170, III:−3.81+/−3.920 cM) [20]. *mor-1* homozygous mutants also have a noticeably rounded mouth and head [21]. Complementation mapping of PAS77 and *mor-1(e1071)* worms showed complete complementation, suggesting that PAS77 is not an allele of *mor-1* (n = 105 progeny counted).

In addition, we selected 39 genes predicted to result in L1 arrest were selected to screen by bacterial feeding RNAi as a method to narrow down the possible loci that could be responsible for the PAS77 mutant phenotype. *glp-1* was used as a positive control for this experiment because it has a dramatic Anterior Pharynx Defective (Aph) phenotype that is easily identified in the PD4792 strain (Figure 2B). Multiple RNAi bacterial strains resulted in pharynx phenotypes (Table 3). The C35D10.5 RNAi resulted in many embryos with a short pharynx phenotype; interestingly, C35D10.5 encodes a predicted ubiquinol cytochrome c reductase assembly protein (Figure 2C). RNAi of M88.2, a mitochondrial ribosomal protein, also produced L1 arrested larvae with a short pharynx phenotype (Figure 2D). While not a PAS77 phenotype, the R74.3 (*xpb-1*) dsRNA bacterial vector resulted in a near-complete absence of *myo-2::GFP* without apparent loss of pharynx muscle as seen in DIC imaging (Figure 2E).

**Table 3:** RNAi Phenotypes observed in candidate genes genetically near PAS136.

Gene Name	Location	Phenotype	Protein Class
*mom-5*	I: 4.12	Pharynx Unattached	Frizzled family of seven transmembrane receptors
*blmp-1*	I: 4.99	Amorphous pharynx	Transcription factor
*sec-8*	I: 5.04	Compressed isthmus	Exocyst complex subunit
*phi-56*	I: 5.06	Anterior bulb/isthmus merged, long, thin procorpus	Signal peptidase subunit
*lam-3*	I: 5.06	Amorphous pharynx	Laminin alpha-2
Y52B11A.10	I: 6.41	Pharynx thin and asymmetric	High osmolarity signaling pathway
*hmr-1*	I: 6.61	Short pharynx	Classical cadherin
*rsr-1*	I: 7.25	Pharynx asymmetry	Splicing co-activator
F56G4.4	I: 7.96	Pharynx asymmetry	Spliceosomal protein

Epitopes recognized by the KT10, KT16, KT17 KT19, KT20 and KT36 antibodies all appeared similar in expression pattern to wild type; the epitopes of these antibodies is unknown however (data not shown) [22]. A test of the MH27 adherens junction antibody (AJM-1) staining showed the presence of normal junctions between epithelial cell types in both wild-type embryos and the pharynx of PAS77 embryos, although all regions of the pharynx are shorter in the anterior-posterior orientation (Figure 2F, G). The MH4 monoclonal antibody to recognize intermediate filament shows that marginal cells are present, and appear similar to wild type (Figure 2H) other than for the compressed shape of the pharynx (Figure 2I).

Like PAS77, the PAS252 phenotype is homozygous recessive and results in an indistinct procorpus and anterior bulb-structure that is more dramatic than PAS77 (compare Figure 1B and 1X). PAS252 worms invariably arrest at the L1 stage, with a short body and tail defects.

The strains PAS120 and PAS154 also share the short pharynx phenotype, however, despite their morphological defects, both are viable and fertile as homozygotes (Figure 1F, K). The larvae of both mutant lines are shorter in length and lethargic in comparison to wild type. They do not appear Dpy; the body size is small, but proportional; however, their heads do not taper toward the mouth as in wild type worms. In both PAS120 and PAS154, the procorpus and isthmus fail to undergo elongation; this effect is more pronounced in the PAS120 strain. Furthermore, both of these phenotypes mapped to a nearly identical region of LG.V. Genetic complementation analysis of PAS120 and PAS154 demonstrate that they are alleles of the same gene (n = 27). Interestingly, *sma-1* maps nearby at 3.54 cM relative to the center of LG.V and encodes a spectrin homolog with a similar pharynx phenotype [23]. Complementation analysis of both PAS120 and PAS154 heterozygous worms with the homozygous *sma-1(e30)* worms produced 50% mutant phenotypes in the F1 generation (n = 99), suggesting these mutant strains represent new *sma-1* alleles. To verify that the *sma-1* locus is altered worms, we sequenced the 17,060 bp region of Chromosome V encoding *sma-1* and found a C to T transition on Chromosome V, genomic position 11905104 [20], which results in the nonsense conversion of a glutamine to a stop codon in the reading frame of the 6^th^ exon in PAS154 *sma-1(lfc1)* worms.

PAS126, PAS129, PAS159, PAS196, and PAS241 also exhibit the short pharynx phenotype; although the primary the defect is a failure for the procorpus to elongate (Figure 1G, H, O, R, W). The PAS126 mutant progeny feed and mature into an adults; however, they are infertile. In contrast PAS196 is homozygous recessive and viable, with a less severe pharyngeal phenotype with a distinct terminal bulb, isthmus, and a partially developed metacarpus. PAS196 worms have a body length of similar size to wild type, although the head is slightly wider. Finally, the PAS241 phenotype results in an extremely wide head and Dpy body with an extremely short pharynx lacking distinct regions (Figure 1W).

### A mutant with pharynx muscle cells that do not appear to adhere

PAS136 mutant progeny exhibit pharyngeal disorganization and misshapen cells in both anterior and posterior portions of the pharynx (Figure 1I). There are also gaps between pharynx muscle cells, which are not normally present. Aside from pharyngeal defects, the organism appears normal but mutant organisms arrest in the L1 stage of development so additional defects are possible. PAS158 and PAS237 display similar phenotypes (Figure 1N, U).

**Figure 3:**
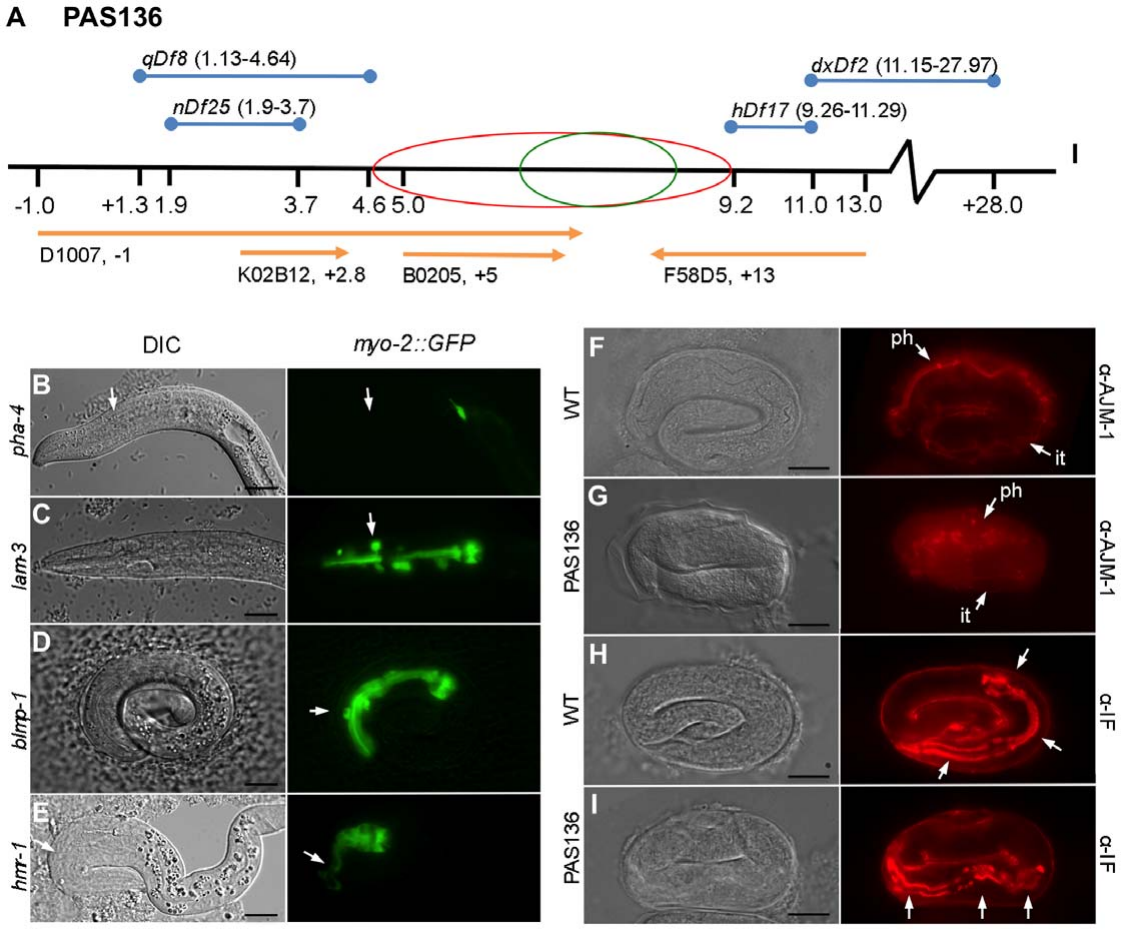
PAS136 mapping and pharynx markers. (A) Probable location of the PAS136 pharynx phenotype allele is between 6 cM and 8 cM on LG.I relative to the genetic center of the chromosome (green circle) derived by mapping with *DraI* or *EcoRI* specific SNPs corresponding to DNA clones D1007, K02B12, B0205, and F58D5 (orange lines) and between 4.64 cM and 9.2 cM (red circle) using complementation with the deficiency strains MT2179, DC1079, KR2838 and SL536 with overlapping chromosomal deletions (blue lines). (B) *pha-4* RNAi used a positive control for pharynx phenotypes, arrow shows lack of *myo-2::GFP* in most of the head. (C) *lam-3* (T22A3.8) RNAi showing a phenotype similar to PAS136 with non-adherent cells (arrow). (D) *blmp-1* (F25D7.3) RNAi has a less severe PAS136 phenotype (arrow denotes cell disconnected from the pharynx). (E) *hmr-1* (W02B9.1) RNAi results in a Pun phenotype with diminished anterior pharynx cells (arrow). (F) Wild-type MH27 AJM-1 adherens junction antibody staining showing pharynx (ph) and intestine (it) localization. (G) PAS136 embryo with weak and disconnect AJM-1 staining in the pharynx (ph) and more normal AJM-1 in the intestine (it). (H) Wild-type Intermediate Filaments showing three sets of marginal cells (arrows). (I) PAS136 embryo with three sets of marginal cells (arrows). Bar is ∼10 µM.

SNP mapping localizes the PAS136 phenotype to LG.I between 1 cM and 8 cM on the right side of the chromosome (Figure 3A, green circle). The combined data from the complementation analyses from deletion strains MT2179, DC1079, KR2838, and SL536 refined the region to be approximately between the 4.64 and 9.25 cM on the right arm of LG.I (Figure 3A, red circle). From these analyses, we have evidence that PAS136 genetically maps between 4.64 cM and 8 cM from the genetic center.

To screen genes in the mapped region as candidates for PAS136, we used RNAi to screen for phenocopy of the pharynx phenotype with bacterial feeding RNAi using the PD4792 *myo-2::GFP* reporter strain. Within the region of PAS136 mapping, there are 322 genes; 39 were chosen for screening using recorded L1 arrest as a necessary parameter; experiments showing pharynx phenotypes are listed (Table 4). A partially penetrant phenotype similar to that of PAS136 resulted from *lam-3* (T22A3.8) RNAi and *blmp-1* (F25D7.3) RNAi (Figure 3C and D); the laminin gene *lam-3* has previously been shown to affect the extracellular matrix and pharynx cohesion, while *blmp-1* is described as a homolog of the B lymphocyte-induced maturation protein 1 on WormBase [24], [25]. Other pharyngeal phenotypes were observed that did not mimic PAS136, such as the Pun phenotypes of *mom-5* (T23F8.1) and *hmr-1* (W02B9.1) (data not shown and Figure 3E).

**Table 4:** RNAi Phenotypes observed in candidate genes genetically near PAS77.

Gene	Location	Phenotype	Protein Class
C26E6.6	III: −2.34	Body morphology variant	
C35D10.5	III: −2.42	Short Pharynx, large head	Ubiquinol cytochrome c reductase assembly protein
*rnp-4*	III: −3.18	Pharynx asymmetry	RNA-binding protein
*dcn-1*	III: −3.19	Body morphology variant	UBA-like ubiquitin ligase
M88.2	III: −3.12	Short Pharynx, Slightly Dpy	Mitochondrial ribosomal protein
*xbp-1*	III: −3.70	*myo-2::GFP* expression limited	bHLH transcription factor
*ccdc-55*	III: −3.72	Pharynx asymmetry	Uncharacterized conserved protein
*wht-3*	III: −3.79	Short Pharynx, Slightly Dpy	ATP-binding cassette (ABC) transporter

The phenotype of *lam-3* RNAi had the most prominent characteristics of the PAS136 phenotype; therefore genetic complementation analysis was conducted using the *lam-3(n2561)* allele in the MT6550 strain. *lam-3* homozygotes are L1 lethal, starved, uncoordinated, and have defective pharyngeal basement membranes. When crossed with PAS136, no F1 phenotypes were seen, although the expected F2 PAS136 phenotype was present in 23% of worms (n = progeny of 6 successful matings). Sequence analysis of the entire 12,963 bp *lam-3* locus of DNA isolated from PAS136 homozygous larva showed no changes from wild type as well.

PAS136 mutants exhibit severe disorganization of the pharynx and appear to have misshapen cells. To investigate the possible lack of adhesion of individual muscle cells, we used the monoclonal antibody MH27 to visualize adherens junctions were present in the mutant organisms. In wild-type worms, the adherens junctions of the pharynx are clearly discernible from those in the hypodermis and other areas because of increase intensity at the buccal cavity, metacorpus and terminal bulb in the wild-type worms (Figure 3F) (n = 11). Mutants did not show any definitive pharyngeal adherens junction staining; however, the adherens junctions of the hypodermis are clearly visible (Figure 3G) (n = 8). Antibody stains of PAS136 with an intermediate filament antibody MH4 revealed marginal cell morphology defects; however, intermediate filaments and marginal cells are clearly present in both wild type and mutants (Figure 3H, I) (WT n = 4, PAS136 n = 4), although not in the normal long, smooth and uniformly shaped morphology of wild-type 3-fold embryos (Figure 3H).

### PAS136 mutants have a wild type number of pharynx muscle cells

The mutant worms of the PAS136 strains appear to have gaps between muscle cells that are not normally present, which could be the result of some muscle cells not be present at all. To determine if all muscle cells were present in the mutant worms, we introduced *a myo-2:GFP:H2B* nuclear reporter gene into the PAS136 strain background. Compared to the organized and consistent wild-type pharynx, the nuclei appeared in random locations throughout the mutant pharynx; however, counts of nuclei showed no significant difference between the numbers of muscle cells seen in the two phenotypes (n = 19 mutant, n = 9 wild type, p = 0.82).

### PAS77 feed better than PAS136 or PAS252

**Figure 4:**
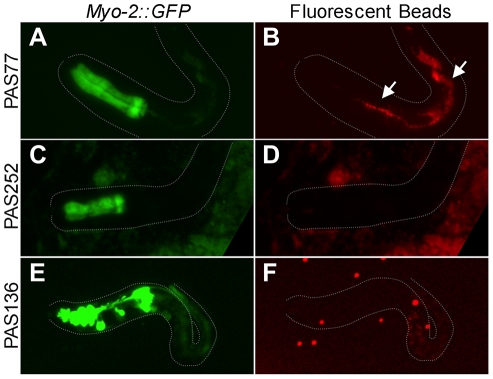
Fluorescent Microsphere Feeding Assay. GFP (left column) and Texas Red (right column) epi-fluorescent photography of live worms after being allowed to feed on fluorescent microspheres mixed with OP50. Dotted lines depict the shape of the worm in each panel. (A) PAS77 mutant phenotype with poorly defined pharynx regions. (B) PAS77 animals are able to ingest fluorescent microspheres (arrows). (C) PAS252 has a similar phenotype as PAS77. (D) PAS252 mutants do not ingest fluorescent microspheres. (E) PAS136 mutant phenotype. (F) PAS136 mutant larvae do not show evidence of ingesting food or beads. Some gut auto-fluorescence is detectable.

To investigate the larval lethality PAS77 and PAS136 progeny, we performed a functional assay using OP50 food mixed with fluorescent beads of approximately the same size as the *E. coli* [26]. PAS77 phenotype worms that fed on Fluoresbrite beads for two hours exhibited beads in both their pharynx and intestine, demonstrating their ability to ingest food normally (Figure 4A, B). However, PAS252 mutant worms with a very similar phenotype did not have beads present in either their pharynx or intestine (Figure 4C, D). Unlike PAS77 worms, in which 12% of worms develop to the L2–L4, PAS252 worms invariably arrest as L1 larvae. The PAS136 phenotype suggests these animals would be incapable to using muscle contractions to ingest food (Figure 4E). Consistent with that hypothesis, no fluorescent beads were found in the intestines of PAS136 larvae (Figure 4F).

### Other pharynx phenotypes

Mutant PAS87, PAS100, PAS117, PAS132, PAS147, PAS155, and PAS197 are among the strains that manifest a tube-like pharynx (Figure 1C, E, J, and not shown). PAS100, which genetically maps to the right arm of chromosome X near 8 cM, may be missing the cells that makeup the procorpus (Figure 1C). PAS117 and PAS155 also exhibit a notched head phenotype (Figure 1E and not shown). PAS147 lacks of a distinct anterior bulb, but otherwise has generally symmetric pharynx morphology (Figure 1J).

The PAS101 strain frequently generated larvae with unattached pharynxes and was mapped between −7 cM and −1 cM on LG.III (Figure 1D). Similarly to PAS101, PAS241 possess a PUN phenotype and the mutant allele is also mapped to LG.III; however, some progeny also demonstrated the short pharynx phenotype (Figure 1V, W).

The PAS117, PAS147, PAS157, PAS158, PAS163, and PAS236 mutant phenotypes show extensive loss of anterior pharynx muscle morphology (Figure 1E, J, L, M, P, T).

## Discussion

The initial goal of this project was to determine the genes involved in posterior pharyngeal muscle fate. Previous studies have shown that the transcription factor, TBX-2, mediates anterior muscle fate. The loss of *tbx-2*, however, does not result in loss of posterior pharyngeal muscle [14], [15]. To address this finding, we carried out a mutagenesis screen using EMS to with the target of isolating the gene or genes responsible for posterior muscle specification. This mutagenesis screen resulted in 83 observed pharyngeal mutant strains of a variety of classes.

Unexpectedly, we were unable to produce any mutants manifesting dramatic posterior muscle loss. We believe that the mechanisms orchestrating posterior muscle fate may occur through functionally redundant genes. Typically, the frequency that a loss-of-function or reduction-of-function allele occurs following EMS mutagenesis is 1/2000 mutagenized *C. elegans* gametes [27]. Therefore, the probability of inducing two loss-of-function alleles, in redundant genes, is 1/4,000,000. Nonetheless, we have managed produce a host mutants yielding morphologically defects in the pharynges.

Surprisingly, 20 of the 83 pharyngeal mutants were homozygous recessive mutants that expressed the short pharynx and rounded mouth phenotype. While the short pharynx phenotype was classified as a pharynx that does not undergo proper pharyngeal elongation; there was variability in the body length among these strains ranging from those with a normal body length to Dpy worms.

We analyzed the short pharynx mutant strain PAS77, which exhibits defects in the metacorpus, procorpus, and manifests a short isthmus. Also, PAS77 usually developmentally arrests and dies during the L1 stage of development, because while the overall proportions of the pharynx are consistent, the length is greatly diminished. Moreover, seeing that the pharynx is essential for the grinding and ingestion of food, it is possible that PAS77 dies due to starvation.

Former research has found that the failure of the isthmus to elongate may be the result of cells not acquiring the proper signals. For instance, *pha-2* mutants (*pha-2* encodes a homeodomain transcription factor protein) manifest a short isthmus [26]. Normally, the region between the metacorpus and terminal bulb have a tight localization of adheren junctions, however worms mutant for *pha-2* show scattered adheren junctions between the metacorpus and the terminal bulb [26]. The inability to form tight junctions within the isthmus suggests that cells are unable undergo correct elongation, ultimately stopping any isthmus growth. However, our mapping of PAS77 shows that it cannot be *pha*-2 because *pha*-2 was mapped to the far left-arm of chromosome X, while PAS77 mapped to chromosome III. It could be possible that these genes, *mor*-1 and *pha*-2, work in a similar genetic pathway.

Interestingly, past research has shown that the *mor-1* gene also results in the rounded pharynx mouth phenotype seen in many of the short pharynx mutants and is located within the same region on chromosome III as PAS77 [21]. While the complementation analysis did not result in a PAS77 phenotype; the *mor-1(e1071)* allele obtained from the *C. elegans* Genetics Center did not display a rounded mouth phenotype, suggesting the strain may not contain a useful allele for complementation studies. Because *mor-1* has not been cloned, it was not possible to test the phenotype by RNAi either.

### The PAS154 mutant phenotype is a result of a new allele of *sma-1*

Through our forward genetic screen we also isolated two other mutants that exhibit similar short pharynx phenotypes: PAS120 and PAS154. Interestingly, in spite of their morphological defect both are viable. Even more, these mutants were chromosomally mapped to chromosome V. Worms with a loss-of-function mutation for *sma*-1 manifest a short pharynx phenotype similar to that of PAS120 and PAS154. In addition, *sma*-1 mutants are viable and map to closely to PAS120 and PAS154 on chromosome V [20].

The *sma*-1 allele encodes a homolog of β-H spectrin, which is a heterodimeric molecule composed of α, β, and βH subunits [23]. In *C. elegans* the βH-spectrin is a part of the cytoskeletal network within the inner cell membrane. βH-spectrin works by providing the cell with structural stability and is required for proper embryogenesis and pharyngeal morphogenesis [28]. Sequence analysis confirmed PAS154 is a novel allele mutant for βH-spectrin, previously documented alleles of *sma-1* share the *sma-1(lfc1)* phenotype of a small body phenotype and short, fat pharynx [23].

### The PAS136 amorphous pharynx phenotype

We hypothesize the extreme disorganization of the pharyngeal muscles and marginal cells of PAS136 homozygous L1 progeny are caused by a mutation in a gene responsible for morphology and cell adhesion. Through genetic mapping techniques including SNP mapping, complementation analysis and RNA interference, the location and function of the PAS136 allele to a 4.6 cM interval on the right arm of Chromosome I, a region with 322 predicted genes. After using RNAi to test 39 genes with predicted L1 lethality in this region, we found a candidate gene, *lam-3*, with a nearly identical phenotype [29]. *lam-3* encodes laminin α-2 ortholog, and has been shown to be integral to remodeling of the extracellular matrix [24]. Although complementation of PAS136 and *lam-3* worms and DNA sequence of the PAS136 *lam-3* allele does not support *lam-3* as the cause of the disorganized pharynx phenotype; it is possible another nearby gene involved in extracellular matrix organization may be involved.

### PAS136 mutant organisms exhibit abnormal pharyngeal adherens junctions

The AJM-1 adherens junction protein that is present in the basal domain of the *C. elegans* apical junction along with DLG-1 [30]. These basal domains of the apical junctions are known to regulate adhesion in the pharynx [31]. The lack of consistent staining AJM-1 antibody in the pharynx of PAS136 embryos suggests that a disruption to the adherence of one cell to another is contributing to the mutant phenotype, specifically in the pharynx since adherens junction staining in other areas of the embryo is nearly normal. This implies that the allele mutated in PAS136 may be specific to the pharynx, although it is possible that AJM-1 or accessory proteins such as DLG-1, LET-413, or HMP-1 are specifically missing pharyngeal enhancers necessary for tissue specific expression1 [30]. In addition, it is likely that there is some redundancy in the proteins involved in adhesion complexes in *C. elegans*. This hypothesis is supported by the *hmp-1*, *hmp-2*, and *hmr-1* mutants that demonstrate a variety of non-identical adhesion defective phenotypes that differ throughout the worm [30], [32]. This suggests that there must be other proteins that are sufficient to maintain the adhesion complexes in the absence of other vital proteins. Since PAS136 appear to only suffer adhesion defects in the pharynx, it is likely the mutation of a gene crucial for pharynx adherens junctions but redundant in those in the hypodermis and other tissues is causing the phenotype.

### PAS136 mutants are not lacking pharynx muscle cells

The *C. elegans* pharynx is made up of exactly twenty muscle cells with 37 distinct nuclei as a result of the fusion of adjacent cells in many muscle groups during development [4]. Counting the nuclei using *myo-2::GFP::H2B* allowed us to determine if the PAS136 mutant worms were missing pharynx muscle cells compared to wild-type worms. There was no significant difference between the number seen in the wild-type worms' pharynxes compared to the mutant pharynxes, indicating that the mutant worms are likely not lacking any *myo-2* expressing muscle cells. As expected, *myo-2::GFP::H2B* nuclei appear randomly positioned in the mutant pharynx as compared to the wild-type pharynx, consistent with the hypothesis that abnormal morphology and adhesion are the key defects in PAS136 animals.

### A genetic screen optimized for rapid identification of pharynx mutant phenotypes

The genetic screen described in this paper was unique in the approach to observing pharynx morphology phenotypes. Because we focused on discovering changes in cell fate or morphology, we used a chromosomally integrated *myo-2::GFP* transgene to facilitate mutant detection using a dissecting microscope equipped with a GFP fluorescence filter. Although alleles of genes such as *pha-1*, *pha-2* and *pha-3*, and *pha-4* were found in previous mutagenesis screens; many of these alleles were isolated in screens that did not focus on pharynx structure [5], [9], [19]. In the case of *pha-1*, the *pha*-1*(e2123)* allele was identified in a screen looking for non-viable eggs at 25°C by visible-light microscopy. Other *pha-1* alleles were isolated by crossing mutagenized male worms with genetically marked *pha-1(e2123)* hermaphrodites [9]. In both cases, the screens were not targeting detecting diverse pharynx phenotypes. The isolation of *pha-2*, *pha-3*, and various Eat mutants was facilitated by using an *unc-31* genetic background to identify slow growing, starved, and abnormally pumping worms under a dissecting microscope [19]; while most pharynx abnormalities will result in slow growth or larval arrest, many genes responsible for these phenotypes are not specific to the pharynx.

*pha-4* alleles have been isolated in numerous screens; although most were not designed to isolate pharynx phenotypes specifically. These include a screen of lethal mutations linked to either *fog-2* or *unc-51 rol-9* [5]; a non-specific L1 lethal screen, and a screen that was looking for deficiencies that suppress the *lin-26* phenotype. In the last study, embryos were stained with the monoclonal antibody MH27 to look for hypodermal defects in a *lin-26* genetic background [33]. Because MH27 stains adherens junctions, which are easily detected in the pharynx, the lack of a pharynx was obvious in the chromosomal deficiency *ozDf2*, which deletes *pha-4* [33]. However, use of adherens junctions antibodies is labor intensive, and the *ajm-1::GFP* expressing strains we have observed do not express strongly and show GFP artifacts. In contrast, the *myo-2::GFP* strain used in our study fluoresced brightly and provided a very distinctive pharynx shape that could be recognized as wild type of mutant immediately.

## Materials and Methods

### Nematode strains and culturing

*C. elegans* strains were obtained from the *Caenorhabditis* Genetics Center (CGC) or colleagues and maintained on NGM plates seeded with the OP50 *E. coli* strain as described in [34], except as indicated for RNAi experiments. The following genetic strains were used in this work: AZ217 (*unc-119(ed3) rulIs37[unc-119(+) partial myo-2 promoter::GFP] III*, [35]), BC4637 (*sDf130(s2427) unc-32(e189) III; sDp3 (III;f)*) [36],BC4697 (*sDf121(s2098) unc-32(e189) III; sDp3 (III;f)*) [36] CB30 (*sma-1(e30) V*) [34], CB1071 (*mor-1(e1071) III*) [21], CB4681 (*nDf17/qC1 dpy-19(e1259) glp-1(q339) III*) [37], CB4856 (Hawaiian isolate) [38], SL536 (*dxDf2/spe-9(eb19) unc-101(m1) I.*), DC1079 (*ces-1(n703) qDf8/hT2[bli-4(e937) let-?(q782) qIs48] (I;III)*), KR2838 (*hDf17/hIn1[unc-54(h1040)] I*), MT2179 (*nDf25/unc-13(e1091) lin-11(n566) I*), MT6550 (*lam-3(n2561)/dpy-5(e61) unc-75(e950) I*) [39], MT690 (*nDf6/unc-93(e1500) dpy-17(e164) III*), MT696 (*nDf12/unc-93(e1500) dpy-17(e164) III*) [40], MT699 (*nDf15/unc-93(e1500) dpy-17(e164) III*) [40], MT6550 *lam-3(n2561)/dpy-5(e61) unc-75(e950) I*), NG2618 (*yDf10 unc-32(e189)/qC1 dpy-19(e1259) glp-1(q339) III*) [41], PD4972 (*mIs11 IV* (*myo-2::GFP*, *pes-10::GFP, gut::GFP*)), SL536 (*dxDf2/spe-9(eb19) unc-101(m1) I*) [42], TY1353. (*yDf10 unc-32(e189)/unc-93(e1500) dpy-17(e164)III*) [41]. All except CB4856 were derived from the reference strain, N2 (Bristol). PAS identifies *C. elegans* strains isolated from an EMS mutagenesis screen.

### Mutagenesis screen

The mutagenesis protocol was modified from Brenner (1974) [34]. PD4972 worms were grown to the L4 stage and washed off plates with M9 buffer solution into 15 mL conical tube. Worms were centrifuged at low speed for 1 minute and the supernatant was removed. The worm pellet was resuspended with 3.0 mL M9 buffer and spun again for 1 minute. 1 mL 0.1 M EMS (Ethyl Methanesulfonate) was administered to the 3.0 mL worm suspension. The conical tube was placed on a rocker for 4 hours at room temperature. Following the mutagenesis protocol, worms were washed twice with M9 buffer and transferred to plates seeded with *E. coli* OP50 in M9 buffer. Healthy looking L4 worms (P0 generation) were picked 1–2 hours post-mutagenesis to new plates and incubated at varying temperatures to control growth periods. PAS77, PAS120, PAS126, PAS136, PAS154, PAS170, and PAS240 were backcrossed 4–8 times with non-mutagenized PD4792 worms.

### SNP mapping

SNP mapping was adapted from Davis et al. with the following modifications [43]. SNP mapping was performed in two phases: chromosome and interval mapping by crossing 10 CB4856 males with 10 heterozygous mutant-line hermaphrodites. After 24 hours, hermaphrodites with copulatory plugs were isolated. For chromosomal mapping, fifty N2 mutant phenotype F2 worms and 50 wild-type F2 worms were picked into separate 1.5 mL tubes, each containing 900 µL worm lysis buffer (50 mM KCl, 10 mM Tris pH 8.3, 2.5 mM MgCl_2_, 0.45% IGEPAL CA-630, 0.45% Tween-20, 0.01% (w/v) gelatin, 60 µg/mL proteinase K. Polymerase Chain Reaction was performed as described in Davis et al. [43] using primers described in that paper.

For interval mapping, the previous protocol was followed with the following exceptions, ninety-six F2 generation phenotypically mutant worms were placed into one well of 96-well PCR plate containing 10 µL of proteinase K (6 mg/mL) diluted 1∶10 with worm lysis buffer. 2 µL of forward primer and reverse primers (100 µM) for specific SNPs were added to 600 µL of the 2X *Taq* Mastermix (NEB) and 600 µL of Nanopure water; reactions of 10 µL of mix and 0.5 µL of individual worm DNA were thermocycled in 96-well plates as described in chromosomal mapping. All primers are identical to Davis et al, 2005 [43] except for the addition of 2.8 LG.I, with the forward primer sequence 5′TCAAATTTGGCACGTCATCAG3′ and reverse primer sequence 5′CTCCATTTTGGAACTCCCAG3′. All regions were cut with *DraI* (NEB) to identify SNPs, except for 2.8 LG.I the DNA was digested *EcoRI-HF* (NEB).

Map positions are measured in centimorgans (cM) and both map positions and physical locations in this article correspond to WormBase release WS226 [20].

### Complementation analysis

Complementation analysis of PAS136 was performed using the following deletion strains: MT2179, DC1079, KR2838, SL536 and MT5990. Known PAS136 heterozygous hermaphrodites were mated with each strain and F1 generation offspring were screened for mutants; verifying that GFP expression and male progeny were present in the F1 generation. For gene identification, PAS120 and PAS154 were mated with CB30; PAS136 was mated with MT6550; and PAS77 was mated with CB1071.

### RNA interference (RNAi)

RNA interference was performed using bacterial feeding RNAi clones from the Ahringer/Geneservice Ltd. feeding library of 16,757 clones [44], [45]. Feeding RNAi was performed as described previously [46] with the following modifications. Bacterial strains were grown 12–16 hours in LB media containing 12.5 mg/mL of tetracycline hydrochloride and 100 mg/mL of ampicillin. Worm plates for RNAi were NGM agar with 0.8 mM IPTG (Amresco) and 150 µg/ml ampicillin (Sigma). 250 µL RNAi bacteria culture was incubated on NGM RNAi plates overnight, and multiple PD4297 L4-stage worms were placed on each feeding plate. After 24 hours, the worms were transferred to a fresh RNAi plate. Progeny were allowed to develop for 1–2 days before phenotypes were scored. Bacteria expressing *pha-4*, *glp-1* or empty pL4440 vector were used as controls for RNAi.

### DNA sequence analysis

DNA from 50 PAS136 mutant L1s, 50 PAS154 mutant L1s, and 50 PD4792 worms was isolated as described in Interval Mapping. PCR products of 24 overlapping sections of the *lam-3* locus in PAS136 and PD4792 and 26 overlapping sections of the *sma-1* locus in PAS154 and PD4792 using custom primers (Table S1 and Table S2) were compared for size on an agarose gel and the PAS136 PCR products were then sequenced in both directions by Functional Biosciences (Madison). Sequence data was assembled into a contig and compared to wild type sequences in WormBase (wormbase.org) [20].

### Feeding assay

Fluoresbrite polychromatic 0.5 µm microspheres (Polysciences, Inc.) were diluted 1∶100 with M9 buffer and 200 µL of the mixture was pipetted onto a 60 mm plate previously seeded with 200 µL of OP50 *E. coli*. The *E. coli* with the Fluoresbrite bead mixture was rubbed gently to mix on the plate. Mutant or wild-type L1 worms were transferred to the plate and allowed two hours to feed. Fed worms were transferred to a 4% agarose pad and photographed using a Zeiss Axiovert 100 microscope using epifluorescence with a Texas Red filter set.

### Immunocytochemistry

Staining with MH4, MH27, KT10, KT16, KT17 KT19, KT20, KT36, and **α**GFP monoclonal antibodies was performed using standard procedures with the following parameters: embryos were incubated in 2% paraformaldehyde in TNB solution [100 mM Tris-HCl (pH 7.5), 150 mM NaCl, 0.5% blocking reagent] for 20 minutes with a cover slip before wicking away excess solution. Slides were frozen on dry ice before quick removal of the coverslip. The slides were then incubated in methanol for 60 seconds followed by a series of acetone washes (modification of [5], [47]). Slides were incubated at 15°C for 12–18 hours using primary antibodies diluted in TNB/NGS: MH3 (1∶3) [12], [48], MH27 (1∶100) [49], KT10, KT16, KT17 KT19, KT20, and KT36 (1∶2–1∶10) [22] and **α**GFP mAB3580 (1∶100) (Chemicon). Slides were washed three times in TBS+0.5% Tween20, before addition of donkey anti-mouse Cy-2 or Cy-3-conjugated antibodies (1∶200 dilution, Jackson ImmunoReseach) for two hours. Slides were washed 3x in TBS+Tween20 and mounted in 50.0% glycerol, 0.006 M Na Citrate, 0.05 M NaH_2_PO_4_, 0.5 mg/ml 4′,6-diamidino-2-phenylindole (DAPI), 25 mg/ml 1,4-diazobicyclo-[2.2.2]-octane (DABCO). The slides were viewed under a Nikon Eclipse TE2000-U microscope and photographed with a Photometrics camera using Metamorph imaging software.

### Pharynx muscle nuclei counts

Nuclearly localized *myo-2::GFP* worms were maintained by microinjecting 0.05 ng/µL *myo-2*::GFP::His2B, 40 ng/µl pRF4, and 60 ng/µl herring sperm DNA into N2 hermaphrodite and screening for rolling progeny. PD4792 males were mated with known heterozygous PAS136 hermaphrodites. The F1 male progeny were mated with *myo-2::GFP::His2B* expressing transgenic worms. After 24 hours the rolling hermaphrodites were moved to individual plates. The F2 offspring of this experiment were screened for mutant organisms possessing *myo-2::GFP*. Wild type and mutants L1 larvae were viewed under the Zeiss Axiovert 100 microscope and photographed in 8–10 vertical sections to record muscle cell nuclei.

### Microscopy

Mutagenized worms were analyzed under a Leica MZ16F compound microscope using Leica EL6000 compact light source. To obtain digital images worms were mounted on 1% agarose pads, with a drop of M9 solution, and covered with a coverslip. Images were acquired using the Motic AE31 microscope with MHG-100B fluorescence attachment and Diagnostic Instruments Spot Camera. Worms were also observed using the Nikon TE2000-U compound microscope using DIC optics or epi-fluorescence to visualize GFP. Metamorph imaging software was used to attain images.

## Supporting Information

Table S1 Oligonucleotide pairs used to amplify and sequence the *lam-3* gene in PAS136.
(DOC)

Table S2 Oligonucleotide pairs used to amplify and sequence the *sma-1*gene in PAS154.
(DOC)
