# Supplementary material for: Multiple Phenotypes Resulting from a Mutagenesis Screen for Pharynx Muscle Mutations in Caenorhabditis elegans
Source: PLoS One. 2011 Nov 2;6(11):e26594. doi: 10.1371/journal.pone.0026594 (PMC3206800; doi:10.1371/journal.pone.0026594)
Supplement: Table S1 — Oligonucleotide pairs used to amplify and sequence the lam-3 gene in PAS136. (DOC) [file pone.0026594.s001.doc]

**Table S1: Oligonucleotide pairs used to amplify and sequence the *lam-3*** gene in PAS136

| **Forward Oligo Name** | **Sequence 5' to 3'** | **Reverse Oligo Name** | **Sequence 5' to 3'** |
| --- | --- | --- | --- |
| lam-3_39_F | AATTTTTCAATGCGGCTCTG | lam-3_799_R | CGAGAAATACGAGAATAAGCGAGA |
| lam-3_414_F | GCATTTTCAGCGCCAATTAT | lam-3_1276_R | GCCATGGCTCATAATATTCTCC |
| lam-3_932_F | GCGACAGATACCTGTGGTCA | lam-3_1726_R | TACCCCGGTAACAGGATCAC |
| lam-3_1433_F | AATGGAAGACCTGGAGCTGA | lam-3_2292_R | CACCGTTCACAATTGAATCCT |
| lam-3_1933_F | TTTTGTTGAGTGGAAAATCAAA | lam-3_2802_R | CCAACAGGGTCACATTCACA |
| lam-3_2436_F | GGCAGGCGTATTGTTGGTAT | lam-3_3302_R | GAAACTATCAGGAGCCTTCCA |
| lam-3_2946_F | GTGTCCTTGCAATCGTGCT | lam-3_4284_R | TCGCAGGAATCTCCTGTTGT |
| lam-3_3939_F | GACACGTCGGAAATCCATCT | lam-3_4764_R | CATCCATCTCCGCTGGTAAT |
| lam-3_4433_F | CGATGCAAAGAGAATCATGG | lam-3_5281_R | TTTGCAAGGACATTCACCAA |
| lam-3_4927_F | TCCTGCTCCAGGACAAGTCT | lam-3_5802_R | TGAAATGTGATGGCTTCTCC |
| lam-3_5446_F | GAAACTTTCATTAGAAGTTGGTTCC | lam-3_6273_R | CAGCTACGTCAAGGGAAACC |
| lam-3_5935_F | GAATTCGACCGGATCAACAG | lam-3_7294_R | TGCTCTGCGAACATCTTGAG |
| lam-3_6950_F | CATTAATGGAGACAATGGATACAA | lam-3_7787_R | TGCTCCACTAAATCTGGCAGT |
| lam-3_7430_F | GGAGGAGCTTTTGAAAATGG | lam-3_8270_R | AACATCTTGGACACGGCTTC |
| lam-3_7915_F | TGGCTGGAAAGTTTGAAAAGA | lam-3_8786_R | GCCATCTTATCAGTTCTTCCTTG |
| lam-3_8415_F | AGATTTGACGGATCGACTGG | lam-3_9265_R | CATCCTTCGAGTTTCCAACG |
| lam-3_8942_F | GCCCATTGCAACAAGTATCA | lam-3_9788_R | CGATCAGCATCTGAATCGAC |
| lam-3_9427_F | ACAACGGGAGAGTGTGAAGG | lam-3_10299_R | TGGATGAGATGATCGGAATG |
| lam-3_9936_F | ACCATCCCAAGTTGGAGAAC | lam-3_10784_R | CTGTCTCCAATTTCCTTGTCG |
| lam-3_10437_F | CAAAAAGTCCATGGAAAAGGA | lam-3_11265_R | AGACCGTATCTAACAGCTCCTTG |
| lam-3_10940_F | CTCATTGGCTCCTGAAAAGC | lam-3_11765_R | TTCAATAAAAATGGGGTGCAG |
| lam-3_11425_F | TCAATGCTTTCAAGGCTCGT | lam-3_12276_R | GAACTTCCAAGGAAAGCAACC |
| lam-3_11941_F | TTGAAAATAGCCGTTCGAAAA | lam-3_12936_R | GCAATCAACAAGGGGCTTT |
| lam-3_11941_F | TTGAAAATAGCCGTTCGAAAA | lam-3_13703_R | GCTTTTTGAGTATTGAGAGTATGAACC |
