# Supplementary material for: Multiple Phenotypes Resulting from a Mutagenesis Screen for Pharynx Muscle Mutations in Caenorhabditis elegans
Source: PLoS One. 2011 Nov 2;6(11):e26594. doi: 10.1371/journal.pone.0026594 (PMC3206800; doi:10.1371/journal.pone.0026594)
Supplement: Table S2 — Oligonucleotide pairs used to amplify and sequence the sma-1gene in PAS154. (DOC) [file pone.0026594.s002.doc]

**Table S2: Oligonucleotide pairs used to amplify and sequence the *sma-1***gene in PAS154

| **Forward Oligo Name** | **Sequence 5' to 3'** | **Reverse Oligo Name** | **Sequence 5' to 3'** |
| --- | --- | --- | --- |
| sma-1_-333_F | TCGATGGATGTCTTCTAATCGTC | ma-1_794_R | CTGGAATCGGAGGATAATCG |
| sma-1_41_F | GCAAGCTACTCGCCTGGAG | sma-1_1143_R | GAATTTGTACGTCGGATGTCGT |
| sma-1_816_F | TGTAATAAACACGGGGAACG | sma-1_1675_R | TCGCCTGATAGTCGTTCTCC |
| sma-1_1319_F | GCTGGATACAATTTCCCGTTT | sma-1_2181_R | TGATCTCCAAATGTTCATGAGT |
| sma-1_1817_F | ACGAAATGCAGGCAAGTTTA | sma-1_3192_R | CCAAGTCACAGTTTTGGAAGTT |
| sma-1_2799_F | GGAAAGTAATGCGGACCAAG | sma-1_4161_R | GGTTGAATTTGGCATGTTCC |
| sma-1_3793_F | TGATGTGGCTGAAAAGAAGC | sma-1_4664_R | CAACTGAGTCCATCGGTCAA |
| sma-1_4308_F | GCTTGGGCACAATTGGATTA | sma-1_5660_R | TGTGGGACATTGACTTCTTCA |
| sma-1_5298_F | AGGGTTGCTGTTGATGCTCT | sma-1_6172_R | TCGTTGGCAATATCGTTGAT |
| sma-1_5829_F | ACTGTCACGCAGACCACTCA | sma-1_6674_R | TGGATGAACATCCTCCATCA |
| sma-1_6323_F | TGAAAGTGCTCGTGAATGGA | sma-1_7156_R | AGAACATCTCTTCGGCGTTG |
| sma-1_6798_F | CCACGAATGTCGAAAGTTGA | sma-1_7678_R | TCGAGTACAGCGTGCTTTTG |
| sma-1_7293_F | AGCGGAGATGATTCAGCATT | sma-1_8145_R | AGCTTTGATTTGGGATGAGG |
| sma-1_7797_F | TGGGCATTACGATGCTGATA | sma-1_8664_R | CGATTGCAGCTCCTTCAAGT |
| sma-1_8295_F | GGCATCGGAAGATTATGGAA | sma-1_9452_R | ACGCGTAATGTTGAGCAGTG |
| sma-1_9097_F | TGGAAGACAACAGACGCTTG | sma-1_9919_R | ATCGATCCATGCCTCAATCT |
| sma-1_9594_F | CGATTGGTTCCAAGAGAACA | sma-1_10438_R | TGTTGCTCTTTTTGGACTTGG |
| sma-1_10100_F | AAGATTGCGAATCGTTGTGA | sma-1_11920_R | CAGTCACCAGTTGTTCTCCTTG |
| sma-1_11567_F | CGATGCTCTTACCAAGAATGC | sma-1_12440_R | AGTCTCCGGATTCACCACTG |
| sma-1_12074_F | TGGCTCGATAAGGTTGAAGG | sma-1_13426_R | TGCTACTCCAGTCGCTTCCT |
| sma-1_13087_F | GAAAGAAAGGTCCGCATCAG | sma-1_13952_R | AGCATTGCCCACGACTTTT |
| sma-1_13574_F | CCAGATACTCGGCACCATCT | sma-1_14443_R | AGCGGTAGTCTTTGCACGAT |
| sma-1_14074_F | CGACTGGAGAATGGTGGATT | sma-1_14917_R | TTGGAGAAGAAGACGGAAGG |
| sma-1_14567_F | ACAGTTGCCCCACTTCAGTC | sma-1_15455_R | AAGCAAAACTAGAAGCAGCACA |
| sma-1_15071_F | TTTGCTTCTCAGGGTTTTCG | sma-1_16428_R | TCTGCCGTCTGGAGTATTCA |
| sma-1_16071_F | TGTGCTAACTCTAACTCTAGTCTCTCA | sma-1_17033_R | GGCCATTTTTAGCACATTGAA |
